# Quantification of enlargement of the levator palpebrae superioris muscle in thyroid eye disease

**DOI:** 10.1007/s10792-026-03967-2

**Published:** 2026-01-27

**Authors:** Lewis Hains, Khizar Rana, Abdullah Almater, Sandy Patel, Dinesh Selva

**Affiliations:** 1 Adelaide Medical School, Adelaide University, Adelaide, Australia; 2 Royal Adelaide Hospital, Adelaide, Australia; 3 Research Excellence Center in Ophthalmology and Visual Sciences, Department of Ophthalmology, College of Medicine, King Saud University, Riyadh, Saudi Arabia; 4 King Saud University Medical City, King Saud University, Riyadh, Saudi Arabia

**Keywords:** Thyroid eye disease, Orbit, Radiology

## Abstract

**Introduction:**

Thyroid eye disease (TED) is an autoimmune-mediated inflammatory condition of the orbit leading to hypertrophy and inflammation of the orbital extra-ocular muscles (EOMs). Previous studies have sought to quantify this enlargement, but due to the anatomical proximity and limitations in imaging quality, have measured the superior rectus (SR) and Levator Palpebrae Superioris (LPS) complex together rather than measuring the individual muscles.

**Methodology:**

Retrospective manual measurements from high-field (3-Tesla) fat-suppressed contrast-enhanced T1-weighted magnetic resonance imaging (MRI) of the orbits were taken from patients diagnosed with TED. To produce a control group, orbital MRIs were measured from patients being investigated for other non-TED related orbital pathology.

**Results:**

47 patients were included with TED with 78 orbits measured (L: 42 R: 37). In the control group, 111 patients with 163 orbits measured were included. In the TED cohort the mean ratio of LPS to SR size was 1.04 (SD: 0.53) compared to 0.947 (SD 0.33) in the non-TED cohort with no statistically significant difference (*p* = 0.3381). The LPS in the TED cohort had a mean muscle thickness of 2.4 mm (SD 0.895), which was higher than the non-TED cohort (*p* < 0.01) which had a mean thickness of 1.56 mm (SD 0.36). This study found LPS enlargement occurred in 48.7% of TED orbits.

**Conclusion:**

This study demonstrates that absolute LPS enlargement is a common finding in TED, occurring in nearly half of affected orbits. This is the first study to individually segment and quantify the extent of enlargement of the LPS relative to the SR using modern medical imaging.

## Introduction

Thyroid eye disease (TED) results from autoimmune-mediated orbital inflammation, commonly associated with conditions such as Graves’ disease (Graves Ophthalmopathy). The exact pathophysiology of TED and its corresponding symptoms is complex and remains poorly understood [1]. Clinical manifestations of TED include proptosis, diplopia and lid retraction and can contribute to significant visual disability [2]. Enlargement and inflammation of the extraocular muscles (EOMs) are a hallmark pathophysiological and radiological feature of TED, and many of the clinical consequences of TED can be largely attributed to this inflammation [3]. Numerous studies have sought to quantify the extent of muscle involvement and enlargement in TED. Traditionally, the inferior rectus and medial rectus muscles are thought to be the most commonly affected EOMs in TED [4]. However, some studies have suggested that the Superior Rectus/Levator Palpebrae Superioris (SR/LPS) complex may be the most severely affected [3]. Whilst the SR and LPS are separate muscle bodies with distinct functions, their close anatomical proximity renders them difficult to distinguish on most previously reported imaging and hence are frequently analysed as a single complex. Few studies have sought to individually separate and quantify the extent of LPS and SR involvement in TED. An improved understanding of the extent of individual muscle involvement may have implications for the clinical manifestations of TED. For example, enlargement of the LPS results in lid retraction, lagophthalmos, and exposure keratopathy that cannot be explained by SR enlargement [5]. This study seeks to individually quantify the extent of LPS and SR involvement in TED.

## Methodology

### Study design

This was a retrospective cross-sectional study conducted in Adelaide, South Australia, using high-field (3-Tesla) fat-suppressed contrast-enhanced T1-weighted magnetic resonance imaging (MRI) of the orbits from patients with and without TED. Patients were retrospectively identified using a local database of TED and non-TED (NTED) patients who had undergone Orbital MRI.

### Ethical approval

This study was approved by the Central Adelaide Local Health Network Human Research Ethics Committee (Approval No. 14279). All elements of the Declaration of Helsinki were adhered to during this study. Due to the retrospective nature of this study, the requirement for informed individual patient consent was waived.

### Study criteria

For the TED group, orbits from patients who had undergone ophthalmological investigation for TED, including an Orbital MRI between May, 2004 and December, 2022, year. All patients had clinical, biochemical and radiological evidence of active TED, diagnosed by oculoplastic surgeons. Patients with inactive or quiescent TED were excluded to avoid heterogenicity. For the control group, orbits from patients who had undergone any form of orbital investigation involving an Orbital MRI. These patients had undergone investigation for various non-TED, pathologies between September 2015 and December 2020 with normal orbital anatomy. Indications for imaging included suspected optic nerve pathology, headache or investigation of contralateral mass lesions. Orbits used for measurements were confided to orbits without structural pathology that would alter normal orbital anatomy. Orbits with bilateral structural disease were excluded.

Patients were excluded if they had orbital disease (e.g., tumors or trauma) or a history of orbital surgery that may have altered normal orbital anatomy. Scans were also excluded if they were of poor quality or not contrast-enhanced, resulting in the inability to reliably differentiate the SR and LPS muscles from adjacent structures. Individuals in the control group with systemic or bilateral orbital diseases, such as IgG4-related disease, were also excluded. For patients with unilateral orbital pathology, only the contralateral, unaffected orbit was included in the analysis.

### Image acquisition and segmentation

**Fig. 1:**
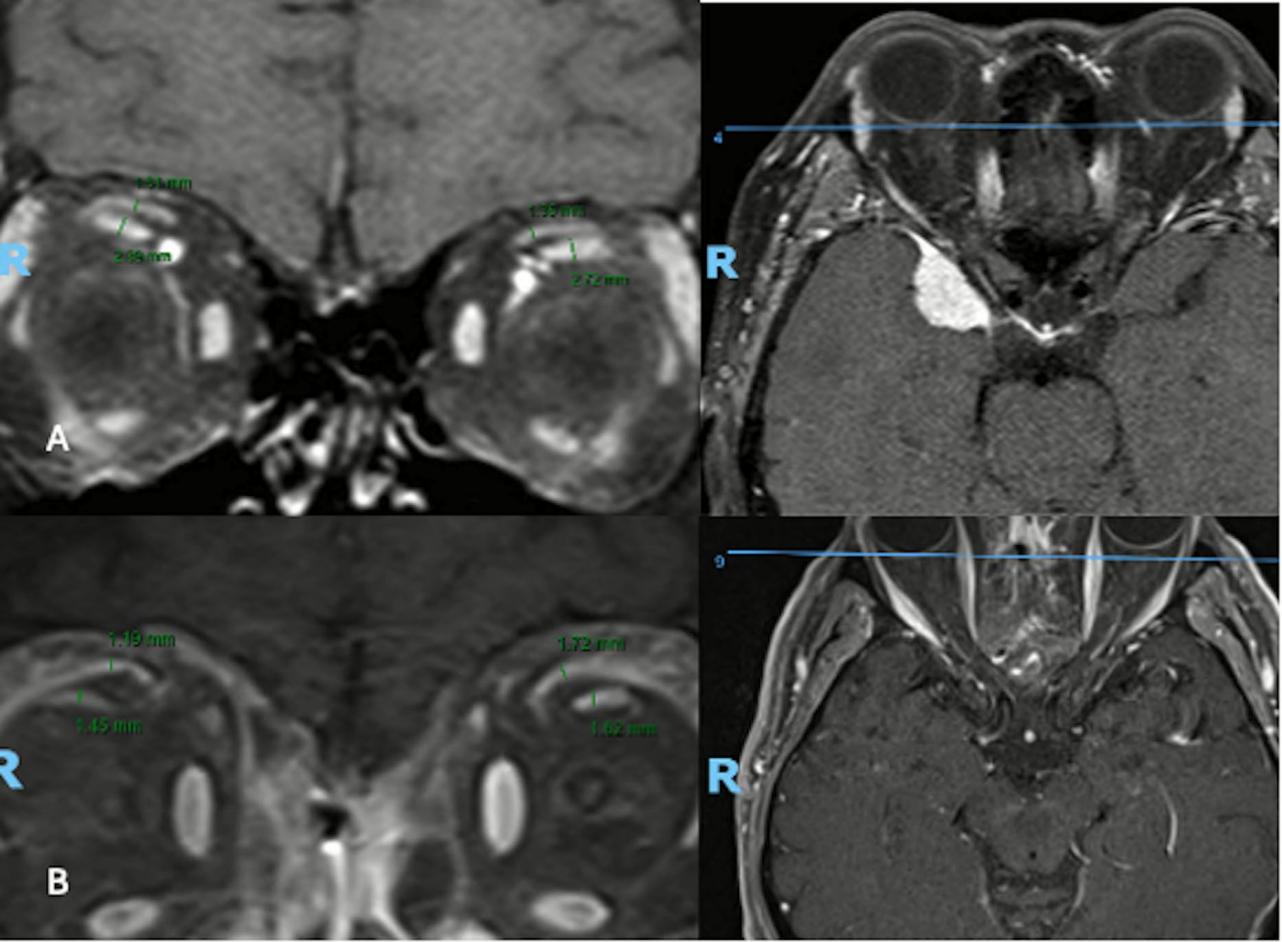
Example of TED orbital measurement with vertical thickness and axial reference image to ensure appropriate position within the orbit. **A** TED Orbit, **B** NTED Orbit)

All imaging was performed using a 3.0 Tesla Magnetom Skyra scanner (Siemens AG, Munich, Germany) utilizing a turbo spin echo sequence (TR/TE: 500/15; field of view: 200 × 200 mm; matrix: 512 × 512; slice thickness: 3 mm). Contrast-enhanced axial and coronal images were acquired following intravenous administration of a standard weight-based dose of gadolinium. Participants were instructed to maintain a forward gaze with gentle eye closure during image acquisition to minimize asymmetric contraction of the extraocular muscles. For each subject, the coronal slice demonstrating the greatest vertical extent of the SR and LPS complex, typically between the mid orbit and orbital apex, was selected for analysis. Muscle thickness was measured manually at the point of maximal muscle belly thickness, and the LPS to SR thickness ratio was calculated for each orbit (Fig. 1). All measurements were performed by a single examiner (L.H) to ensure consistency in segmentation technique. Measurements were the examiner expressed uncertainty regarding measurements were validated by a second author (A.A).

### Statistical analysis

Statistical analysis was performed using RStudio (version 2025.05.1 + 513). Individual measurements of the LPS and SR muscles were taken as above and a ratio of LPS to SR size was derived as a marker of relative LPS enlargement. Shapiro–Wilk tests were used to assess normality for each variable within both the TED and non-TED cohorts. All variables were found to have deviated significantly from the normal distribution (*p* < 0.05), thus requiring non-parametric statistical methods for group comparisons. Wilcoxon rank-sum (aka Mann–Whitney U test) testing was used to compare changes in LPS size, SR size and LPS/SR ratio changes between the TED and non-TED cohorts. LPS and SR size testing had prespecified directionality (one-sided) due to the hypothesis that TED would result in muscle enlargement. For the LPS/SR ratio, two-sided testing was used given a lack of directional expectation regarding relative muscle involvement. Spearman’s rank correlation was used to assesss association between age LPS and SR size and the LPS/SR ratio. Sex differences were evaluated using the Wilcoxon rank-sum test. Paired Wilcoxon signed-rank tests were used to compare measurements between the left and right orbits.

To identify orbits with disproportionate LPS enlargement, a threshold of two standard deviations above the mean LPS/SR ratio in the non-TED cohort was used as a cutoff (1.61). Similarly, orbits with LPS or SR sizes that exceeded two standard deviations above the mean NTED control were identified. TED orbits exceeding this threshold were classified as having isolated LPS enlargement and their frequency was reported. This was also replicated for the SR EOM, using 2 standard deviations below the non-TED LPS/SR ratio mean (0.287).

## Results

Forty-seven patients with active TED were included in the study. This accounted for 79 orbits with a mean age of 57.37 (SD 15.97), 33 patients (70.2%) were female. In the control (non-TED) cohort, 111 patients were included with 163 orbits measured. The mean age for the non-TED cohort was 57.79 (SD 17.65) and 62 (55.8%) were female (Tables 1 and 2).

**Table 1:** Baseline patient characteristics and measurements between TED and non-TED cohorts

	TED	Non-TED
Number of patients/orbits	47/79	111/163
Age, mean (SD)	57.37 [15.97]	57.69 [17.65]
Number of Female Patients (%)	33 (70.2)	62 (55.8)
Levator Palpebrae Superioris (LPS) Height mm, mean [SD]	2.4 [0.895]	1.56 [0.36]
Superior Rectus (SR) Height mm, mean [SD]	2.53 [0.865]	1.77 [0.542]
LPS/SR Ratio, mean [SD]	1.04 [0.532]	0.947 [0.33]

*TED thyroid eye disease, *SD* standard deviation

**Table 2:** Statistical comparisons between muscle groups and patient characteristics

Variable	Cohort	LPS	SR	LPS/SR Ratio
Age (Spearman’s ρ, p)	TED	0.758, 0.509	-0.259, 0.822	0.078, 0.493
	NTED	-0.288, < 0.05	0.181, < 0.05	-0.385, < 0.05
Gender (M vs F) (p value)	TED	0.184	0.147	0.066
	NTED	0.387	0.211	0.648
Laterality (L vs R) (p value)	TED	0.869	0.305	0.352
	NTED	0.776	0.596	0.339

The mean ratio of LPS size to SR size in the TED cohort was 1.04 (SD 0.532) vs 0.947 (SD 0.33) with no statistically significant increase in the LPS/SR ratio between the two cohorts (*p* = 0.3381) suggesting proportional enlargement in TED. The LPS in the TED cohort was larger than compared to the non-TED cohort (*p* < 0.001) with mean thickness of 2.4 mm (SD 0.895) compared to 1.56 mm (SD 0.36). Similarly, the SR thickness in the TED cohort was larger than in the non-TED cohort (*P* < 0.001) with a mean thickness of 2.53 mm (SD 0.865) vs 1.77 (SD 0.542). No significant correlation was identified between age and muscle enlargement in the TED cohort (LPS *p* = 0.509, SR *p* = 0.822, LPS/SR ratio *p* = 0.493). However statistically significant correlations were observed in the non-TED cohort, with the LPS being negatively correlated with age (ρ = –0.288, *p* < 0.001), and SR positively correlated with age (ρ = 0.181, *p* = 0.021). The LPS/SR ratio showed a strong negative correlation with age (ρ = –0.385, *p* < 0.001). No statistically significant difference was observed between sex and muscle sizes in both the TED and NTED cohorts (*p* > 0.05). No difference was identified between left or right orbits and muscle sizes in both cohorts (*p* > 0.05).

**Fig. 2:**
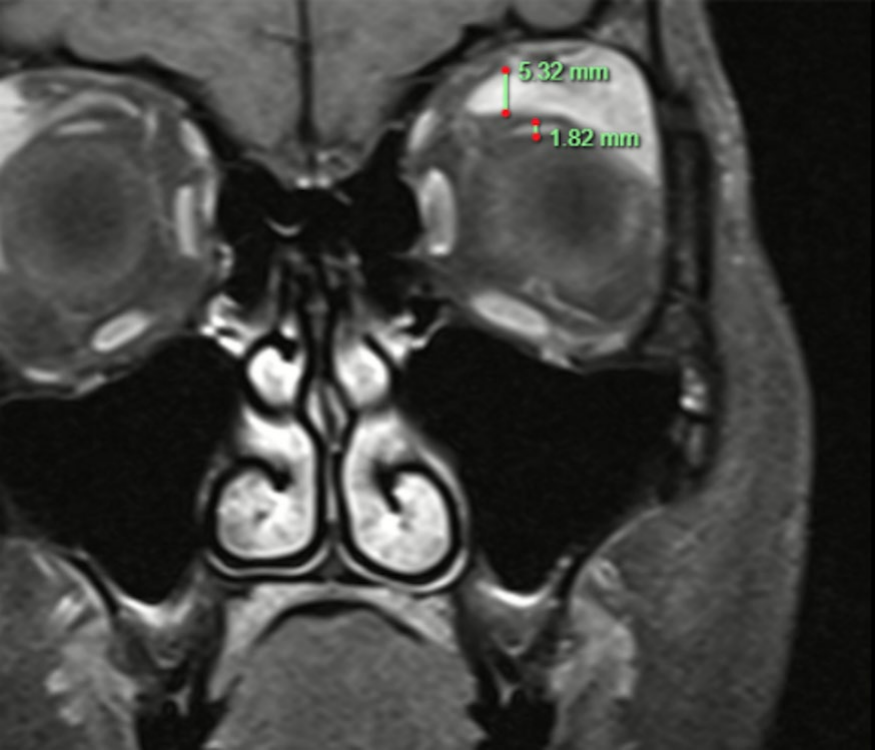
Example of disproportionate left LPS hypertrophy in TED (LPS/SR Ratio 2.92)

**Fig. 3:**
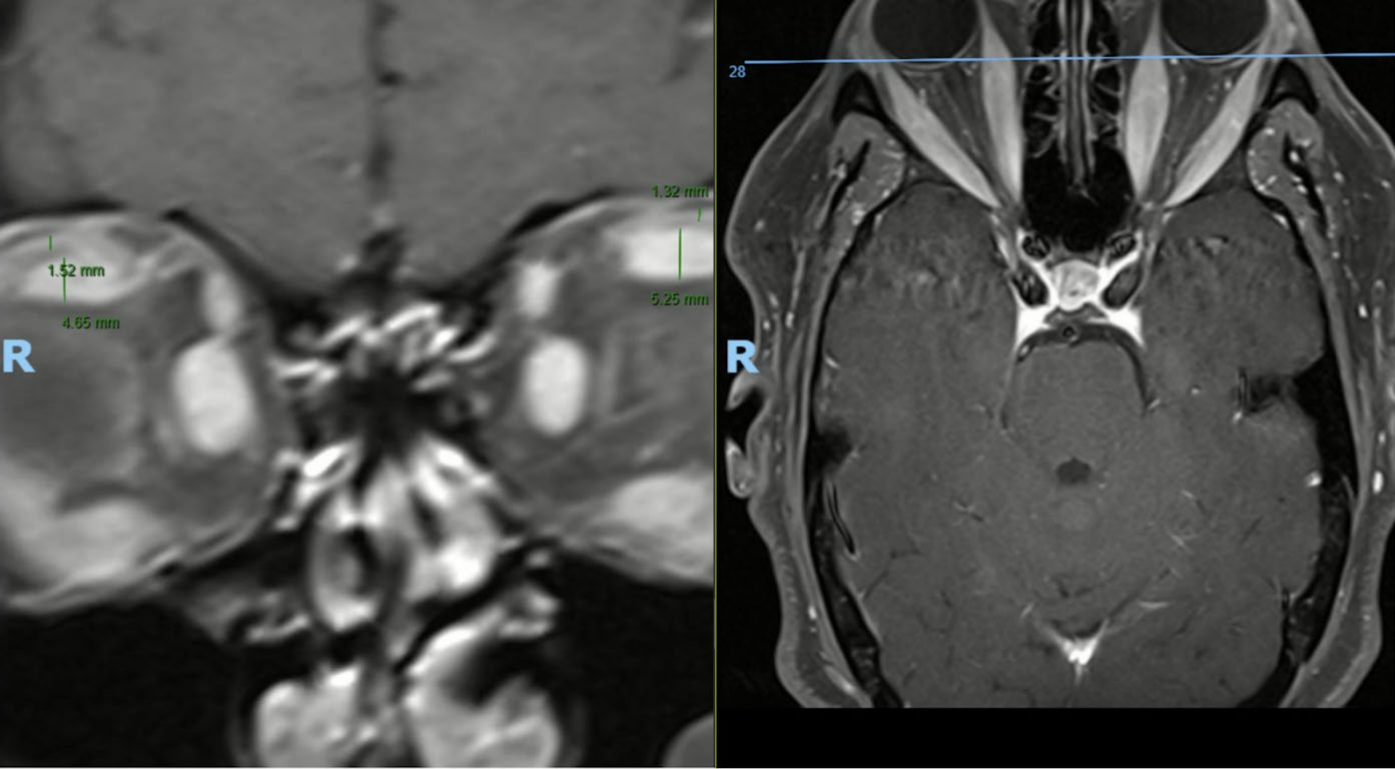
Example of disproportionate left SR hypertrophy in TED (LPS/SR ratio 0.251) with similar disproportionate enlargement in the right orbit, however, this was less than 2 standard deviations from the control mean

LPS enlargement (defined as > 2 standard deviations above the NTED mean) was observed in 38 orbits (48.7%) and 22 orbits had enlargement of the SR (28.2%). Thirteen (16.67%) orbits had concurrent SR and LPS enlargement. Nine TED orbits (11.5%) demonstrated discordant LPS/SR ratios greater than 2 standard deviations above the non-TED mean indicating selective LPS predominance in a subset of cases (Fig. 2). Conversely, only 1 orbit (1.28%) was identified with isolated SR enlargement (Fig. 3).

## Discussion

This is one of the first studies to individually quantify the extent of SR and LPS muscle involvement in TED using modern, 3 T MRI. In cases of disproportionate LPS/SR size ratios, individual delineation of the muscles may correlate with symptom severity and may explain cases of significant lid retraction without significant vertical diplopia and vice versa. In this case, absolute hypertrophy was seen in both the LPS and SR EOMs but favoured the LPS.

Our reported muscle thicknesses are largely concordant with other studies reporting individual muscle thickness in active TED. Liu et al. reported similar enlargement of the LPS (whilst not measuring the SR), with maximal muscle thickness of 2.18 mm in active TED patient cohorts and 1.24 mm in the control groups [6]. Our study also builds on the work of Ohnishi et al. who first utilised sagittal MRI to demonstrate absolute LPS enlargement in active TED and correlated this with upper eyelid retraction. Whilst this older study used 0.5-Tesla imaging to measure absolute thickness, our study uses modern high resolution 3-Tesla imaging and individually segmented the LPS alongside the SR to propose a novel metric in the LPS/SR ratio. This ratio has the potential to help normalise for inter-patient anatomic variability and successfully identifies subsets of patients with isolated muscle enlargement discordant to its muscle complex counterpart. This study also reported LPS size as a potential tool for quantitative evaluation of active TED. Other volumetric studies have also identified statistically significant enlargement of the SR in TED versus health control cohorts [7]. When measured as a conjoined complex using 3-dimensional computerised tomography based, volumetric analysis, the LPS/SR complex has been identified to occur in 54% of patients with TED compared to 16.6% in our study [3].

Traditionally, the inferior rectus muscle is taught as the most commonly affected muscle in active TED [8]. More recent studies have proposed that the LPS/SR complex may be more severely affected [3]. The precise mechanism as to why preferential muscle involvement occurs in TED remains poorly understood. Broadly, molecular mechanisms involving thyrotropin receptor autoantibodies and increased expression of thyrotropin receptor in the orbital tissue of TED patients have been proposed as key mechanisms of autoimmunity in the orbital tissue [8].

This study has several limitations. Firstly, the retrospective nature of this study introduces inherent limitations and reduces the generalisability of these findings. Similarly, the small sample size, particularly in the TED cohort, may affect the generalisability of these findings. Given the close anatomical proximity of these muscles and potential for overlap on coronal MRI may contribute to potential segmentation errors. Despite efforts to select the most appropriate coronal slice and ensure consistent measurement technique, variability in image quality and subjective interpretation may have affected the precision of these measurements. To mitigate these concerns, measurements where the author expressed concern about the accuracy of these given measurements were reviewed. The non-TED cohort may also be subject to selection bias, and given the various indications for orbital MRI, such as contralateral mass lesions or optic nerve pathology, does not represent a truly healthy control cohort. As a result, subtle orbital changes may have influenced these measurements. Whilst not directly reported or assessed in this study, assessment the extent of enlargement of the individual muscles may offer further clinical correlation. Cases with heterogeneous enlargement of the EOMs, as demonstrated in our study, may favour certain forms of surgical management. Routine radiological assessment of the LPS/SR ratio may offer the ability to help further personalise surgical options toward patient presentations. Furthermore, measuring the LPS and SR size individually may potentially have use as a radiological means of differentiating TED phenotypes in lid-dominant and muscle-dominant disease. Previous studies have demonstrated a positive correlation between upper eyelid retraction and the size of the LPS [9, 10]. In contrast to the work by Ohnishi et al., our study did not report standardised clinical grading of features of TED such as lid-lag, eyelid retraction or strabismus. Hence, this study was unable to directly correlate discordant muscle enlargement with symptom severity or specific TED clinical phenotypes. Instead our study demonstrates that description of the muscle complex as a ratio may be more appropriate in order to identify specific muscle-predominant disease and challenges recent volumetric studies which measure these muscles as a complex. As mentioned previously, many of these studies differ in that they have relied on measurements of the entire LPS/SR complex as a surrogate of LPS size when measuring at the more posterior aspects of the orbit to demonstrate these relationships.

Future studies should seek to use automated segmentation tools to individually segment and determine the precise muscle volume and/or size of the LPS/SR complex. Correlation of these findings with clinical features such as lid retraction scores and other disease activity metrics may strengthen these findings and highlight the importance of individual muscle segmentation.

## Data Availability

The datasets generated and/or analysed during the current study are not publicly available due to ethical considerations but are available from the corresponding author on reasonable request.
